# Construction of a High-Performance Composite Solid Electrolyte Through In-Situ Polymerization within a Self-Supported Porous Garnet Framework

**DOI:** 10.1007/s40820-023-01294-0

**Published:** 2024-01-12

**Authors:** An-Giang Nguyen, Min-Ho Lee, Jaekook Kim, Chan-Jin Park

**Affiliations:** Department of Materials Science and Engineering, Chonnam National University, 77 Yongbong-ro, Buk-gu, Gwangju, 61186 South Korea

**Keywords:** Scalable tape-casting method, Self-supported porous Li_6.4_La_3_Zr_1.4_Ta_0.6_O_12_, Composite solid electrolyte, LiF-and B-rich interphase layers

## Abstract

Composite solid electrolytes (CSEs) have emerged as promising candidates for safe and high-energy–density solid-state lithium metal batteries (SSLMBs). However, concurrently achieving exceptional ionic conductivity and interface compatibility between the electrolyte and electrode presents a significant challenge in the development of high-performance CSEs for SSLMBs. To overcome these challenges, we present a method involving the in-situ polymerization of a monomer within a self-supported porous Li_6.4_La_3_Zr_1.4_Ta_0.6_O_12_ (LLZT) to produce the CSE. The synergy of the continuous conductive LLZT network, well-organized polymer, and their interface can enhance the ionic conductivity of the CSE at room temperature. Furthermore, the in-situ polymerization process can also construct the integration and compatibility of the solid electrolyte–solid electrode interface. The synthesized CSE exhibited a high ionic conductivity of 1.117 mS cm^−1^, a significant lithium transference number of 0.627, and exhibited electrochemical stability up to 5.06 V vs. Li/Li^+^ at 30 °C. Moreover, the Li|CSE|LiNi_0.8_Co_0.1_Mn_0.1_O_2_ cell delivered a discharge capacity of 105.1 mAh g^−1^ after 400 cycles at 0.5 C and 30 °C, corresponding to a capacity retention of 61%. This methodology could be extended to a variety of ceramic, polymer electrolytes, or battery systems, thereby offering a viable strategy to improve the electrochemical properties of CSEs for high-energy–density SSLMBs.

**Supplementary Information:**

The online version contains supplementary material available at 10.1007/s40820-023-01294-0.

## Highlights

- A scalable tape-casting method produces self-supported porous Li_6.4_La_3_Zr_1.4_Ta_0.6_O_12_.
- Combining the in-situ polymerization approach, a composite solid electrolyte with superior electrochemical properties is fabricated.
- Solid-state Li|CSE|LiNi_0.8_Co_0.1_Mn_0.1_O_2_ cells show remarkable cyclability and rate capability.
- LiF-and B-rich interphase layers mitigate interfacial reactions, enhancing solid-state battery performance.

**Supplementary Information:**

The online version contains supplementary material available at 10.1007/s40820-023-01294-0.

## Graphical abstract

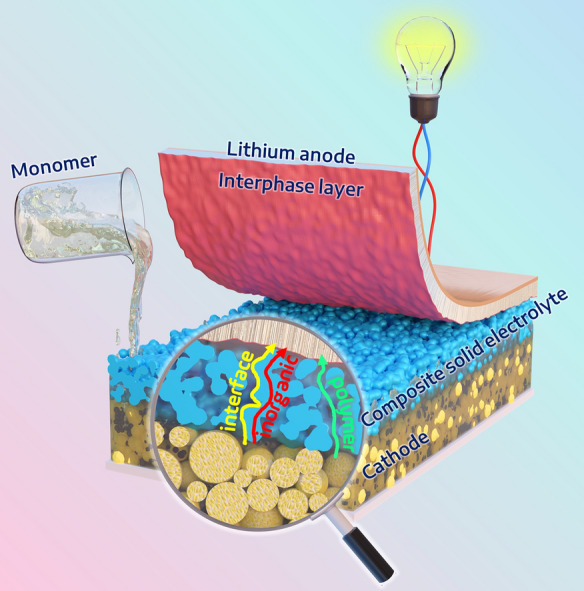

## Introduction

The modern landscape is rapidly shifting from wired to wireless connections across a wide range of products. As a result, rechargeable batteries are becoming essential for powering electric vehicles and electronic devices [1, 2]. Consequently, several types of next-generation rechargeable batteries, such as solid-state lithium metal batteries (SSLMBs) [3, 4], lithium-sulfur batteries [5, 6], sodium-ion batteries [7], and potassium-ion batteries [1, 8–10] have been explored. Among them, SSLMBs offer potential as high-energy–density rechargeable batteries due to the low redox potential of –3.04 V *vs.* standard hydrogen electrode and the high theoretical specific capacity of 3860 mAh g^−1^ from lithium metal anode [3, 4, 11, 12]. In the context of SSLMBs, the solid electrolyte serves as a crucial component, profoundly influencing the overall performance of the batteries. Therefore, over the past decades, several types of solid electrolytes have been explored in SSLMBs, including inorganic solid electrolytes, solid polymer electrolytes (SPEs), and composite solid electrolytes (CSEs) [13, 14]. Traditional inorganic solid electrolytes such as garnet [15, 16], sodium super ionic conductor [17], perovskite [18], sulfide [16, 19, 20], and halide [21, 22] exhibit good ionic conductivity (> 10^–4^ S cm^−1^) at room temperature. Their brittle nature and poor contact with electrodes, however, can lead to large interfacial resistance, limiting the overall performance of SSLMBs [13, 14]. Polymer electrolytes are economical, flexible, and scalable, yet often fall short in ionic conductivity at room temperature [13, 14, 23, 24].

To address these limitations, CSEs have gained attention for their ability to merge the benefits of both inorganic and polymer-based SSLMBs [25–28]. Numerous strategies involving the addition of 0D, 1D, and 2D fillers into a polymer matrix have been proposed over the years [13, 14]. Unfortunately, these approaches often lead to the random distribution of fillers, resulting in crossing junctions, discontinuous networks, and agglomeration which adversely affect their electrochemical properties [14]. Therefore, it is essential to design strategies that create continuous pathways for Li^+^ diffusion and prevent filler agglomeration to significantly enhance ionic conductivity.

Additionally, the interface contact between solid electrodes and electrolytes can lead to interfacial incompatibility and increased internal resistance, negatively affecting the electrochemical properties of SSLMBs [13, 14]. To mitigate this limitation, in-situ polymerization is proposed as a promising approach [29–31]. This method involves the injection of liquid monomer precursors into batteries to facilitate and establish interfacial contact. Following injection, a polymerization reaction is initiated to convert the monomers into polymer electrolytes. This process integrates the solid electrolyte—solid electrodes interface, significantly reducing interfacial resistance [29–32].

Motivated by these considerations, this study successfully developed a CSE through the combination of self-supported porous Li_6.4_La_3_Zr_1.4_Ta_0.6_O_12_ (LLZT) and in-situ polymerization into the LLZT. The resulting CSE exhibited a unique and integrated structure, comprising continuous inorganic, polymer, and inorganic-polymer interface pathways to promote ion transport. Remarkably, the CSE achieved a high ionic conductivity of 1.117 mS cm^–1^ and a high lithium transference number of 0.627, remaining stable up to 5.06 V *vs.* Li/Li^+^ at 30 °C. Thanks to the synergistic combination of the well-designed CSE and the formation of LiF and B-rich interphase layers, the solid-state Li|CSE|LiNi_0.8_Co_0.1_Mn_0.1_O_2_ cell remained a discharge capacity of 105.1 mAh g^–1^ after 400 cycles at 0.5 C and 30 °C, corresponding to a capacity retention of 61%. This strategy suggests a novel approach for designing fast ionic conductor electrolytes for high-energy SSLMBs.

## Experimental Procedure

### Preparation of Composite Solid Electrolyte

The self-supported porous Li_6.4_La_3_Zr_1.4_Ta_0.6_O_12_ (LLZT) was prepared using a tape-casting method, as detailed in our previous report [31]. Briefly, LLZT, povidone as a surfactant, poly(vinyl butyral) as a binder, benzyl butyl phthalate as a plasticizer, and starch as a porous agent were mixed in a solution containing ethanol and acetone (5:5 in vol%) in the following mass ratios: 100:5:10:20:20, respectively. Furthermore, to counterbalance the Li depletion in LLZT during the subsequent calcination phase, 10 wt% lithium nitrate was incorporated into the aforementioned mixture. Subsequently, the slurry was cast on nylon tape by using a doctor blade and dried at 80 °C. Afterward, the dried tape was cut into a 19 mm circle shape, followed by peeled-off the nylon tape. Then, the self-supported porous LLZT film was obtained by heating the above samples at 1100 °C for 1 h. The thickness of the LLZT film was tried to maintain at 100 ± 5 μm. Solutions containing monomers were meticulously prepared by incorporating a precise ratio of polyethylene glycol methyl ether methacrylate (MEMA) monomer, succinonitrile (SN), fluoroethylene carbonate (FEC) plasticizer, and a mixture of lithium bis(trifluoromethanesulfonyl)imide (LiTFSI) and lithium difluoro(oxalato)borate (LiDFOB) salts. The molar ratio of LiTFSI to LiDFOB was fixed at an optimized ratio of 19:1, according to previous reports [33, 34]. In addition, FEC was kept at 5 wt%. Subsequently, 1.0 wt% of azobisisobutyronitrile (AIBN) as a thermal initiator was incorporated into the above solution. Thereafter, 20 µL of this solution was injected thrice into the self-supported porous LLZT framework, followed by a heating process at 80 °C for 12 h to initiate polymerization. This sequence resulted in the formation of the CSE. For comparative purposes, the solid polymer electrolyte (SPE) was synthesized using a method analogous to that of the CSE, with the primary distinction being the substitution of Whatman® glass fiber in place of the self-supported porous LLZT.

### Material Characterization

The crystal structures of the materials were determined using an X-ray diffractometer (XRD; Empyrean, Malvern Panalytical) equipped with a PIXcel^3D^ detector and a Cu anode (λ = 1.5406 Å). The thermal behavior of the composites was studied using a thermogravimetric analyzer (TGA–50, Shimadzu). The molecular structures of the materials were identified by a Fourier transform infrared spectrometer (FTIR; Spectrum 400, PerkinElmer). A field emission scanning electron microscope (FE–SEM; Gemini 500, Zeiss) and a transmission electron microscope (TEM; JEM-2100F, Jeol) were used to investigate the morphology of the samples. To estimate the conversion ratio of the monomer, the monomer or polymer was dissolved in deuterated dimethyl sulfoxide (DMSO-d6) and analyzed using a nuclear magnetic resonance (NMR) spectrometer (Advance III HD 400, Bruker) operating at 400 MHz. Solid-state NMR analysis was performed using an ECZ400R spectrometer (Jeol) at 9.4 T (400 MHz). The chemical state of materials was examined using an X-ray photoelectron spectrometer (XPS; K–Alpha^+^, Thermo Scientific). The binding energies were calibrated based on the C 1*s* peak (284.8 eV). The surface chemistry of electrodes was characterized utilizing Time-of-Flight Secondary Ion Mass Spectrometry 5 (TOF–SIMS 5; ION-TOF GmbH). Bi_3_ primary ions, operating at 30 keV and 0.97 pA, were directed towards a 100 × 100 μm^2^ analysis area on the solid surface of the electrodes. Following ion bombardment, the resulting secondary ions were collected and subsequently documented by a detector operating at a resolution of 128 × 128 pixels throughout the data acquisition process. For the depth profile, a 1 keV Cesium ion beam was employed to sputter an area of 300 × 300 μm^2^.

### Electrochemical Characterization

Electrochemical characterization was conducted using CR2032-type coin cells, with stainless steel (SS) serving as a blocking electrode. The electrochemical impedance spectroscopy (EIS) of the electrolytes was analyzed using SS|electrolyte|SS symmetric cells across a frequency range of 100 kHz–1 Hz with an applied voltage amplitude of 10 mV via a potentiostat/impedance analyzer (Zive SP2, WonATech). Ionic conductivity ($\sigma$) was measured at different temperatures to compute the activation energy ($E_{a}$) of the electrolyte using Eq. (1):

1 $$\sigma = A^{{\frac{{{-}E_{a} }}{RT}}}$$

where *R* represents the gas constant, *A* is the pre-exponential constant, and *T* is the temperature.

Linear sweep voltammetry was performed to assess the electrochemical potential stability of the electrolytes in Li|electrolyte|SS asymmetric cells from open circuit potential to 6 V *vs.* Li/Li^+^ at a scan rate of 1 mV s^−1^ and temperature of 30 °C. A single-step chronoamperometry technique was applied to calculate the Li-ion transference number ($t_{{Li^{ + } }}$) using Li|electrolyte|Li symmetric cells with an overpotential of 10 mV at 30 °C, as per the Bruce and Vincent method [14]. The plating/stripping of the electrolyte in Li|electrolyte|Li symmetric cells were analyzed to assess the stability of the electrolyte against Li metal using a battery cycler (WBCS 3000, WonATech).

Electrodes composed of LiNi_0.8_Co_0.1_Mn_0.1_O_2_ (NCM811) were prepared via a casting method using a doctor blade. A slurry composed of 70 wt% NCM811 active materials, 20 wt% Super P carbon (Timcal), and 10 wt% polyvinylidene difluoride in N-Methyl-2-pyrrolidone solvent was spread onto an aluminum current collector and dried in a vacuum oven at 80 °C for 12 h. The dried foil was cut into circles with a 14 mm diameter, and the loading mass of the active material was controlled at 1.5 mg cm^−2^. To fabricate a Li|CSE|NCM811 cell, the NCM811 cathode was impregnated with a 5 µL solution of LiTFSI:LiDFOB:SN in a molar ratio of 0.95:0.05:20 at 80 °C. Subsequently, the self-supported porous LLZT framework was inserted, followed by the injection of 20 µL of a monomer-containing solution thrice. Afterward, a 16 mm Li chip was then inserted to construct the Li|CSE|NCM811 cells. The cell was then subjected to heating at 80 °C for 12 h to facilitate in-situ polymerization.

Galvanostatic charge–discharge tests were performed using the battery cycler within a potential range of 2.5 − 4.3 V *vs.* Li/Li^+^ at 30 °C. The C-rates were calculated based on the practical specific capacity (200 mAh g^–1^) of the NCM811 cathode.

### Calculation Method

Density Functional Theory calculations were performed using the generalized gradient approximation in the Heyd–Scuseria–Ernzerhof exchange–correlation functional as implemented in the Quantum Espresso package [35–37]. Structure relaxation was executed using the Broyden–Fletcher–Goldfarb–Shanno algorithm, setting the convergence thresholds for energy and force to less than 10^−5^ Ry and 10^−5^ Ry/Bohr, respectively. Data visualization was accomplished through the utilization of the VESTA and XcrySDen software tools [38, 39].

## Results and Discussion

### Material Characterization

**Fig. 1:**
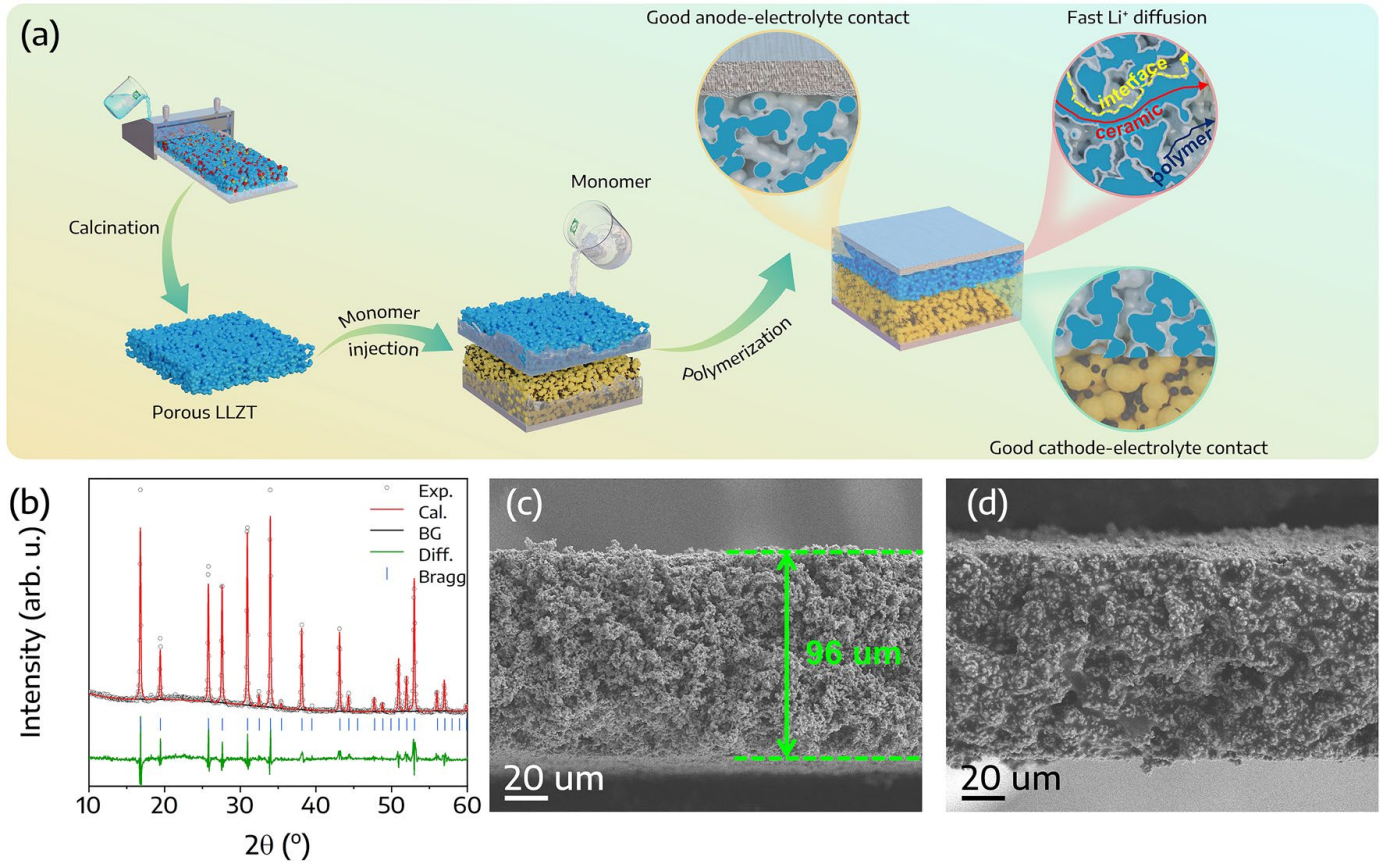
**a** Schematic representation of the CSE fabrication process. **b** Rietveld refinement of the XRD pattern for the self-supported porous LLZT. SEM images of **c** self-supported porous LLZT and **d** CSE

A CSE with a self-supported porous LLZT structure was prepared as illustrated in Fig. 1a. The self-supported porous LLZT structure was prepared via the tape-casting method, as detailed in the Experimental section. Following this, a monomer solution composed of lithium bis(trifluoromethanesulfonyl)imide (LiTFSI) and lithium difluoro(oxalato)borate (LiDFOB) salts, succinonitrile (SN) and fluoroethylene carbonate (FEC) as plasticizers, and polyethylene glycol methyl ether methacrylate (MEMA) monomer at a desired ratio, was injected into this structure. This was subsequently in-situ polymerized to construct the CSE. LiDFOB was chosen for its ability to generate interphase layers on electrodes and its superior solubility compared to its analogues (e.g., lithium bis(oxalato)borate) [40]. Additionally, FEC was used to reduce the reactivity of SN towards the Li metal. Meanwhile, the self-supported porous LLZT structure was designed to prevent LLZT particle agglomeration and facilitate Li^+^ transport through the ceramic, polymer, and their interface pathways. Concurrently, in-situ polymerization aids in the formation of a beneficial interface between the electrolyte and both electrodes.

The crystallographic structure of the self-supported porous LLZT was analyzed using XRD. As shown in Fig. 1b, all diffraction peaks corresponded accurately to the LLZT cubic structure without any trace of impurity phase (COD #96-155-2157) [41]. Within the LLZT unit cell, La^3+^ occupies the 24c position with eightfold coordination, while Ta^4+^ and Zr^4+^ are situated at the 16a position in an octahedral arrangement. Both 24d and 94h sites contain Li, while O resides at the 96h sites (Fig. S1). These details are exhibited in Fig. 1b and Table S1. It has been reported that the cubic structure of LLZT enhances lithium conductivity at room temperature [16, 42]. Additionally, images of the self-supported porous LLZT film and CSE can be found in Fig. S2. The integration of the polymer rendered the originally white LLZT film partially translucent. Furthermore, the XRD pattern of CSE can be found in Fig. S3. The pattern confirms that CSE preserves the inherent cubic structure of the self-supported porous LLZT. A minor peak at 28.5° is attributable to SN. Notably, after a 10-day exposure to ambient conditions, with temperatures varying from 20 to 30 °C and relative humidity levels between 50 and 70%, the structure of CSE exhibited minimal alterations, underscoring its stability in ambient atmospheric conditions.

In the cross-sectional SEM image (Fig. 1c), the self-supported porous LLZT presents a thickness of ~ 96 μm, forming a continuous 3D conductive network. Subsequently, the MEMA monomer was permeated and underwent in-situ polymerization within the remaining voids of the structure (Fig. 1d). The presence of a continuous interface between the LLZT and the polymer can accelerate Li^+^ diffusion. Additionally, the in-situ polymer might also enhance the interface with solid electrodes.

**Fig. 2:**
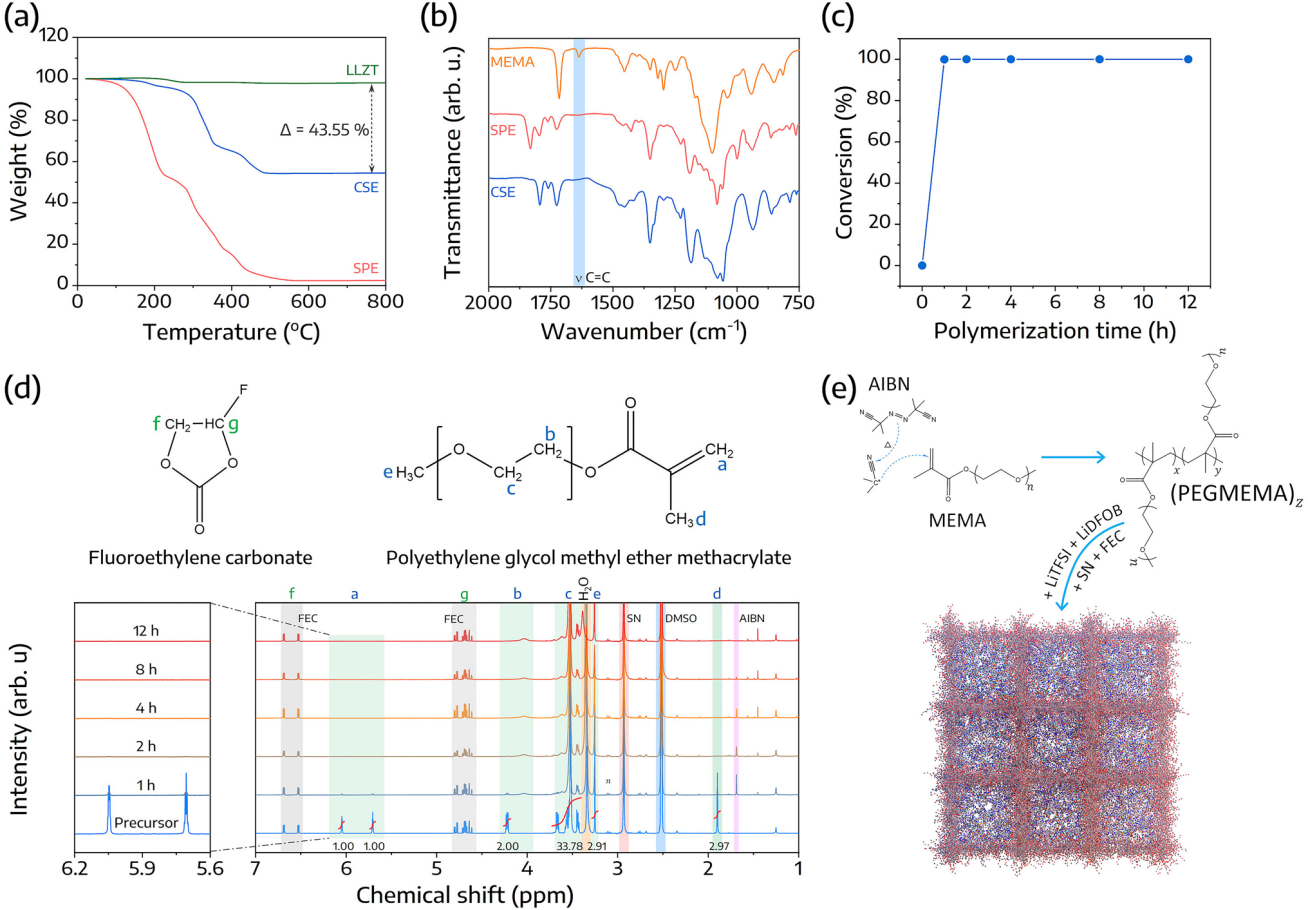
**a** TGA curves for LLZT, SPE, and CSE. **b** FTIR spectra for MEMA, SPE, and CSE. **c** Conversion rates of the MEMA-based monomer. **d** Structures and 1H NMR spectra of the precursor at various heat treatment times from 0 to 12 h. **e** Suggested polymerization reaction mechanism

Moreover, the percentage of LLZT in the CSE was determined based on the TGA results, as presented in Fig. 2a. The TGA profile for CSE displayed two weight reduction stages. The initial phase, ranging from 30 to 350 °C, corresponds to SN and LiDFOB decomposition, whereas the second phase, from 350 to 490 °C, associates with the decomposition of LiTFSI (Fig. S4). A total weight loss of 45.76 wt% was observed for the CSE. Comparatively, the weight percentage of LLZT slightly decreased by 2.21%. The weight loss difference of 43.55% between LLZT and CSE can be attributed to the organic compounds present in the CSE. The residual 56.45 wt% is attributable to the inorganic component of LLZT. Furthermore, the CSE exhibited thermal stability up to a temperature of 200 °C, registering a weight loss of less than 3 wt%. Additionally, the glass transition temperature (*T*_*g*_) for both SPE and CSE was determined through DSC. Figure S5 reveals that the *T*_*g*_ values for SPE and CSE are − 54.49 and − 63.79 °C, respectively. The notably lower *T*_*g*_ of CSE can be primarily attributed to the presence of self-supported porous LLZT and the ion–dipole interactions between Li^+^ ions and the polar groups within the polymer [43]. Such factors promote increased segmental mobility in the polymer, suggesting enhanced ionic conductivity for CSE.

The polymerization mechanism of the MEMA monomer was investigated via FTIR. As observed in Fig. 2b, the infrared absorption peak positioned at approximately 1636 cm^−1^ is assigned to the C=C in the MEMA monomer. However, following in-situ polymerization, these absorption peaks disappeared, signifying that polymerization of the MEMA occurred. It is also noteworthy that the initial fluid state of the precursor solution transformed into a solid state without any fluidity after polymerization, as displayed in Fig. S6. Moreover, the conversion rate was estimated by comparing the integrals of ^1^H signals from hydrogen bonded to C=C with ^1^H signals of the polymer from the NMR spectra, as illustrated in Fig. 2c [44]. Typically, the proton signals of the precursor were deconvoluted (Fig. 2d). Peaks at 5.71 and 6.05 ppm correspond to the signal of hydrogen from =CH_2_, which significantly reduced after a polymerization time of 1 h, corresponding to a conversion rate of 99.92%. As the polymerization period was extended, these peaks disappeared, indicating the full polymerization of MEMA. However, the characteristic peak of the AIBN initiator at 1.70 ppm still existed and was only eliminated when the reaction time reached 12 h. Therefore, to prevent any residual initiator, the polymerization time was maintained at 12 h. Furthermore, based on the FTIR and NMR results, the polymerization reaction is proposed to be a thermal free-radical polymerization (Fig. 2e). Upon heating, the AIBN initiator could release free-radicals, which could potentially attack the C=C bond in MEMA, resulting in its polymerization. Subsequently, the resultant polymer is able to capture other components such as salts and plasticizers.

### Electrochemical Characterization

**Fig. 3:**
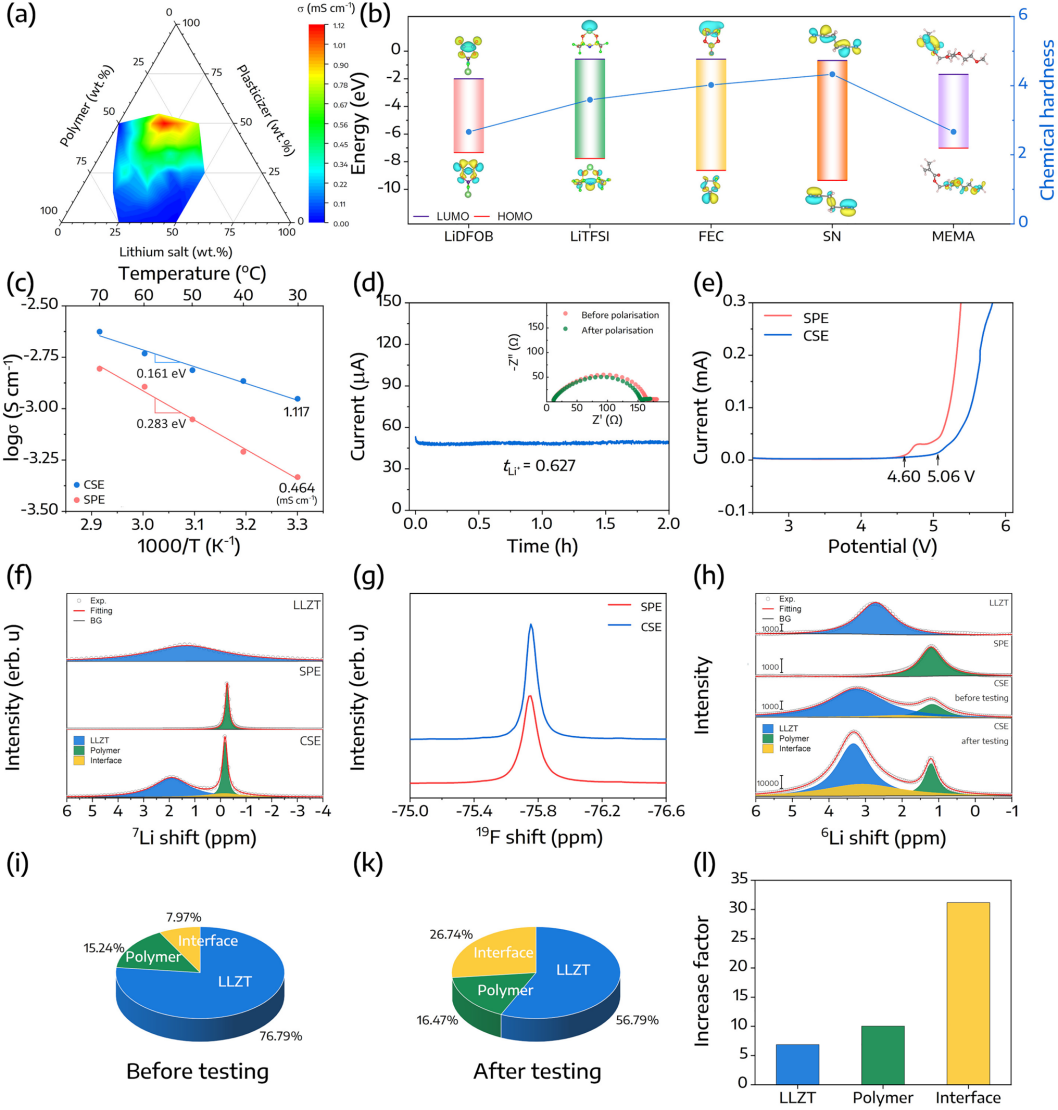
**a** Ionic conductivities of CSE depicted on the ternary phase diagram at 30 °C. **b** LUMO and HOMO energy values of LiDFOB, LiTFSI, FEC, SN, and MEMA. **c** Arrhenius plots illustrate the conductivity of CSE and SPE. **d** Current transient profile along with corresponding EIS plots for Li|CSE|Li symmetric cell, both before and after polarization. **e** Linear sweep voltammograms of CSE and SPE. **f** ^7^Li and **g** ^19^F solid-state NMR spectra of LLZT, SPE, and CSE. **h** ^6^Li solid-state NMR spectra of LLZT, SPE, and SE before and after testing. Contribution of LLZT, polymer, and their interface **i** before and **k** after polarization. **l** Increase factor of the intensity of LLZT, polymer, and their interface

A ternary diagram was employed to optimize the lithium ionic conductivity ($\sigma$) of the CSE and to elucidate the influence of its constituent components on the conductivity tendency. The compositions of the electrolytes and their respective ionic conductivities at 30 °C are listed in Table S2. Typically, the maximum ionic conductivity of 1.117 mS cm^−1^ can be achieved at a MEMA:SN:lithium salts ratio of 30:50:20, as illustrated in Fig. 3a. It is important to note that an increased percentage of SN as a plasticizer might enhance the ionic conductivity, but it can also render the organic component fluid due to its eutectic mixture with FEC [33]. Thus, for subsequent investigations, a MEMA:SN:lithium salts ratio of 30:50:20 was employed.

Furthermore, the frontier molecular orbital energies of the salts, plasticizers, and polymer were evaluated to provide additional insight into their reduction and oxidation behaviors. Electrons in the highest occupied molecular orbital (HOMO) are more likely to be donated, which leads to lower oxidation potential stability of the molecule. In contrast, the lowest unoccupied molecular orbital (LUMO) possesses a strong electron affinity and correlates with reductions in higher-potential regions. As displayed in Fig. 3b, LiDFOB exhibited the lowest LUMO energy level as well as the highest HOMO energy level. Besides, the average energy gap between LUMO and HOMO, also known as chemical hardness ($n$), can also serve as an indicator of the molecule’s relative reactivity [45]. Given that LiDFOB has the lowest $n$ value of 2.66, it implies that LiDFOB is most susceptible to decomposition. This suggests that LiDFOB can undergo reduction on the anode side and oxidation on the cathode side, functioning as a dual-role film-forming additive, thereby leading to the formation of LiF and B–rich interphase layers. Further examination of these phenomena will be conducted through XPS and TOF–SIMS techniques. In contrast, SN exhibited a broad LUMO–HOMO gap accompanied by the highest $n$ value, indicating its superior thermodynamic stability.

The ionic conductivity of the CSE was measured over a temperature range of 30 to 70 °C. In parallel, the ionic conductivity of the SPE was also investigated for comparative analysis. As indicated in Fig. 3c, the ionic conductivity of the CSE was 1.117 mS cm^−1^, superior to that of SPE, which was only 0.464 mS cm^−1^ at 30 °C. Moreover, the activation energy ($E_{a}$) for the CSE was calculated to be 0.161 eV, while the SPE recorded an $E_{a}$ value of 0.283 eV.

Another key parameter for evaluating solid electrolyte materials is the lithium transference number ($t_{{Li^{ + } }}$). To determine this parameter, the chronoamperometry technique was utilized in lithium symmetric Li|CSE or SPE|Li cells at 30 °C (Figs. 3d and S7). Accordingly, the calculated $t_{{Li^{ + } }}$ of CSE was 0.627, which is significantly higher than the value of 0.236 observed for SPE. This finding infers that the CSE facilitates more efficient and faster transportation of Li^+^ ions. Besides, the enhancement in electrochemical properties can be ascribed to the self-supporting porous LLZT matrix, which facilitates Li^+^ ion conduction, consequently shortening their conduction pathway. This can be largely credited to the synergistic interplay between the long-range, continuous conductive porous LLZT framework, the aligned polymer matrices, and the solid-polymer interface, all synergistically contributing to the proficient transport of Li^+^ ions. Furthermore, asymmetric Li|SPE or CSE|stainless-steel cells were fabricated to study the electrochemical stability potential of the electrolytes with a scan rate of 1 mV s^−1^ at 30 °C. Based on the linear sweep voltammetry (LSV) results shown in Fig. 3e, the CSE maintained stability up to 5.06 V *vs.* Li/Li^+^, while the SPE started to exhibit oxidative tendencies above 4.60 V. The remarkable electrochemical stability observed can be attributed to the presence of LLZT ceramic in CSE, which is known for its high oxidative stability [28, 46, 47].

Solid-state NMR is a valuable tool for exploring the chemical environments and mobility of Li^+^ within solid electrolytes. Therefore, this method was employed to gain a deeper understanding of the improvement in the transference number and ionic conductivity of CSE. In Fig. 3f, the ^7^Li solid-state NMR spectra for the LLZT, SPE, and CSE are presented. The ^7^Li peak in LLZT and SPE was located at − 0.27 and 1.40 ppm, respectively. However, in CSE, these peaks were downfield shift to − 0.17 and 1.92 ppm, respectively. Moreover, an interface between LLZT and CSE was also formed, as displayed in Fig. 3f. This suggests that Li^+^ experienced deshielding, resulting in reduced electronic density and weaker interactions with both the polymer and LLZT, thereby facilitating their mobility [43]. Furthermore, the ^19^F peak of LLZT exhibited an upfield shift and higher intensity compared to that of SPE (Fig. 3g). It means the F^–^ were shielded and engaged in interactions with the polymer or LLZT, hindering their transport. Consequently, this contributes to the reduction of the anion transference number as well as the enhancement of the Li^+^ transference number [43].

Besides, the contribution of each component in CSE was identified using ^6^Li isotopic-labelled process. On the Earth, two stable isotopes, ^6^Li and ^7^Li, are found with natural abundances of 7.5% and 92.5%, respectively. Therefore, in any lithium-containing compound (e.g., CSE), ^7^Li is predominantly present. Through the application of an external potential to the ^6^Li|CSE|^6^Li cell, ^6^Li^+^ ions pass through and gradually substitute ^7^Li^+^ ions in the CSE. This process led to the enrichment of the active components of CSE with ^6^Li. By analyzing the solid-state NMR of ^6^Li before and after testing, we could elucidate the contributions of LLZT, polymer, and their interface [30, 31, 48–50]. Typically, the ^6^Li peaks for LLZT and SPE were observed at 2.72 and 1.21 ppm, respectively, as depicted in Fig. 3h. Subsequently, the ^6^Li peak of CSE, both before and after testing, was deconvoluted into three peaks corresponding to the LLZT phase, polymer phase, and their interface. Based on the areas under the fitting curves, the contributions of the LLZT phase, polymer phase, and their interface in CSE before testing were 76.79%, 15.24%, and 7.97%, respectively (Fig. 3i and Table S3). After testing, these values changed to 56.79%, 16.47%, and 26.74%, respectively (Fig. 3k and Table S3). Furthermore, the increase factor could be calculated based on the area ratio of each phase before and after testing. As illustrated in Fig. 3l, the increase factor for LLZT, polymer, and their interface was determined to be 6.87, 10.04, and 31.18, respectively. These results strongly suggest that the LLZT-polymer interface serves as the most favorable route for Li^+^ movement.

**Fig. 4:**
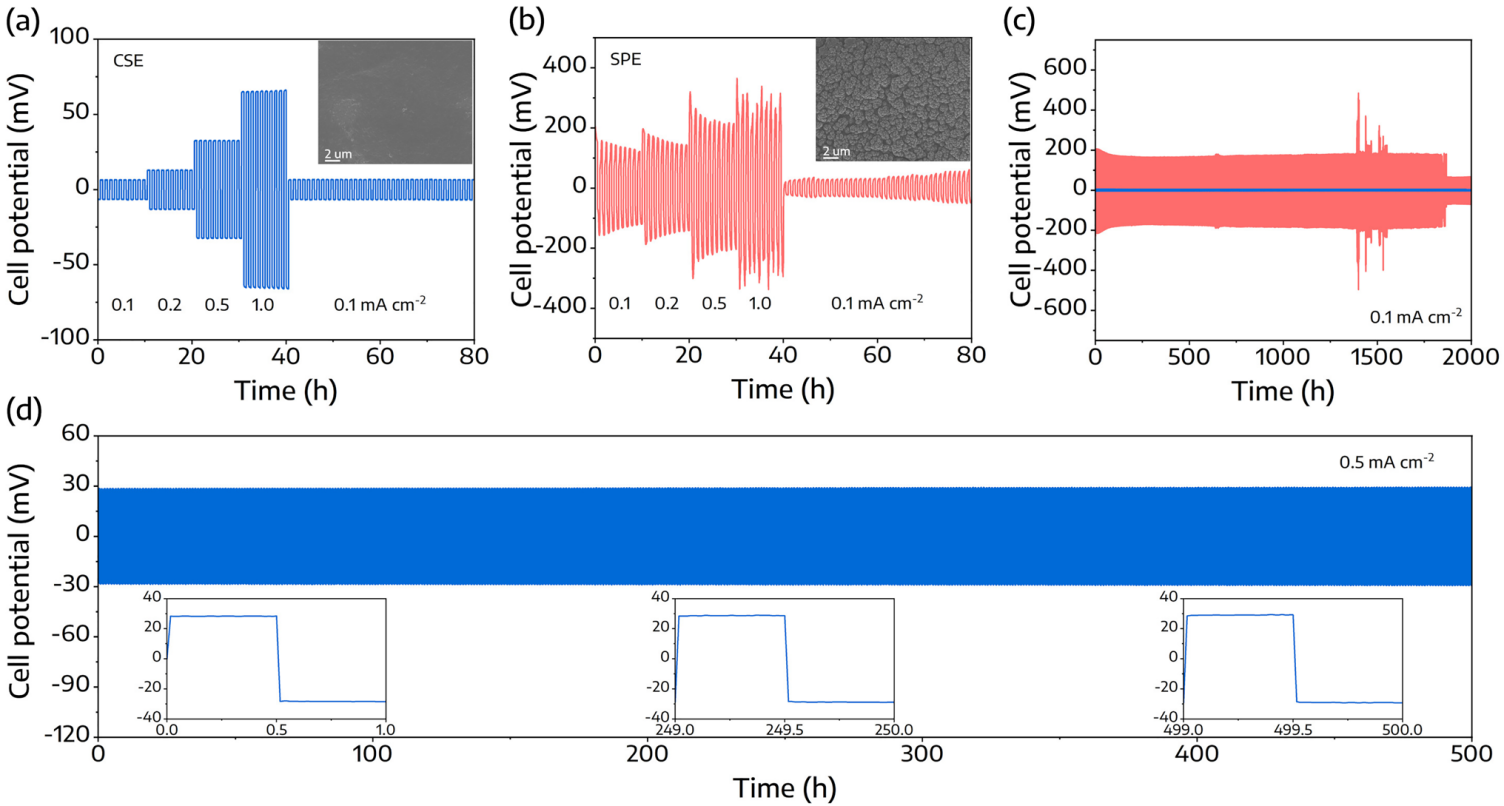
Rate capabilities of **a** Li|CSE|Li and **b** Li|SPE|Li symmetric cells. **c** Long-term cycling performance of Li|CSE or SPE|Li symmetric cells at a current density of 0.1 mA cm^−2^. **d** Long-term cycling performance of Li|CSE|Li symmetric cells at a current density of 0.5 mA cm^−2^, conducted at 30 °C

The stability of the CSE against lithium metal was explored via a galvanostatic lithium stripping/plating test, executed at 30 °C using a symmetric Li|CSE|Li cell. First, the lithium stripping/plating was carried out for 10 cycles at various current densities from 0.1 to 1.0 mA cm^−2^, as represented in Fig. 4a, b. Notably, the Li|CSE|Li symmetric cell yielded overpotentials of 6.5, 12.7, 32.2, and 65.1 mV at current densities of 0.1, 0.2, 0.5, and 1.0 mA cm^−2^, respectively. Remarkably, upon reducing the current density back to 0.1 mV cm^−2^, the Li|CSE|Li cell still remained square-wave-like curves with a negligible overpotential of 6.6 mV. On the other hand, the Li|SPE|Li cell displayed sawtooth-wave-like curves with significant polarization. To further emphasize the superior characteristics of CSE, lithium from cycled cells was retrieved for SEM analysis, as shown in Fig. 4a, b insets. Cycled lithium metal from Li|CSE|Li cells exhibited a uniform, smooth surface, whereas dendrite-like morphologies were observed on the lithium metal retrieved from Li|SPE|Li cells. These observations suggest that the CSE structure promotes uniform lithium deposition.

Additionally, the long-term interfacial stability of the CSE at 0.1 mA cm^−2^ was examined and represented in Fig. 4c. The Li|CSE|Li cell exhibited consistent square-wave-like curves with a steady overpotential of approximately 5.0 mV over 2000 h, showing no signs of short-circuiting. In sharp contrast, the Li|SPE|Li cell displayed a significant overpotential of approximately 170 mV, with unstable plating/stripping behavior after 1500 h and an apparent short-circuit after 1900 h. Moreover, at a higher current density of 0.5 mA cm^−2^, the Li|CSE|Li symmetric cell demonstrated outstanding plating and stripping performance, maintaining a negligible overpotential of approximately 29.5 mV over 500 h, as displayed in Fig. 4d. These results establish that symmetric Li cells incorporating CSE offer superior long-term cycling stability, even under high current densities. In essence, the CSE demonstrated superior stability in contact with Li metal, suggesting its potential as a solid electrolyte for high energy density SSLMBs.

**Fig. 5:**
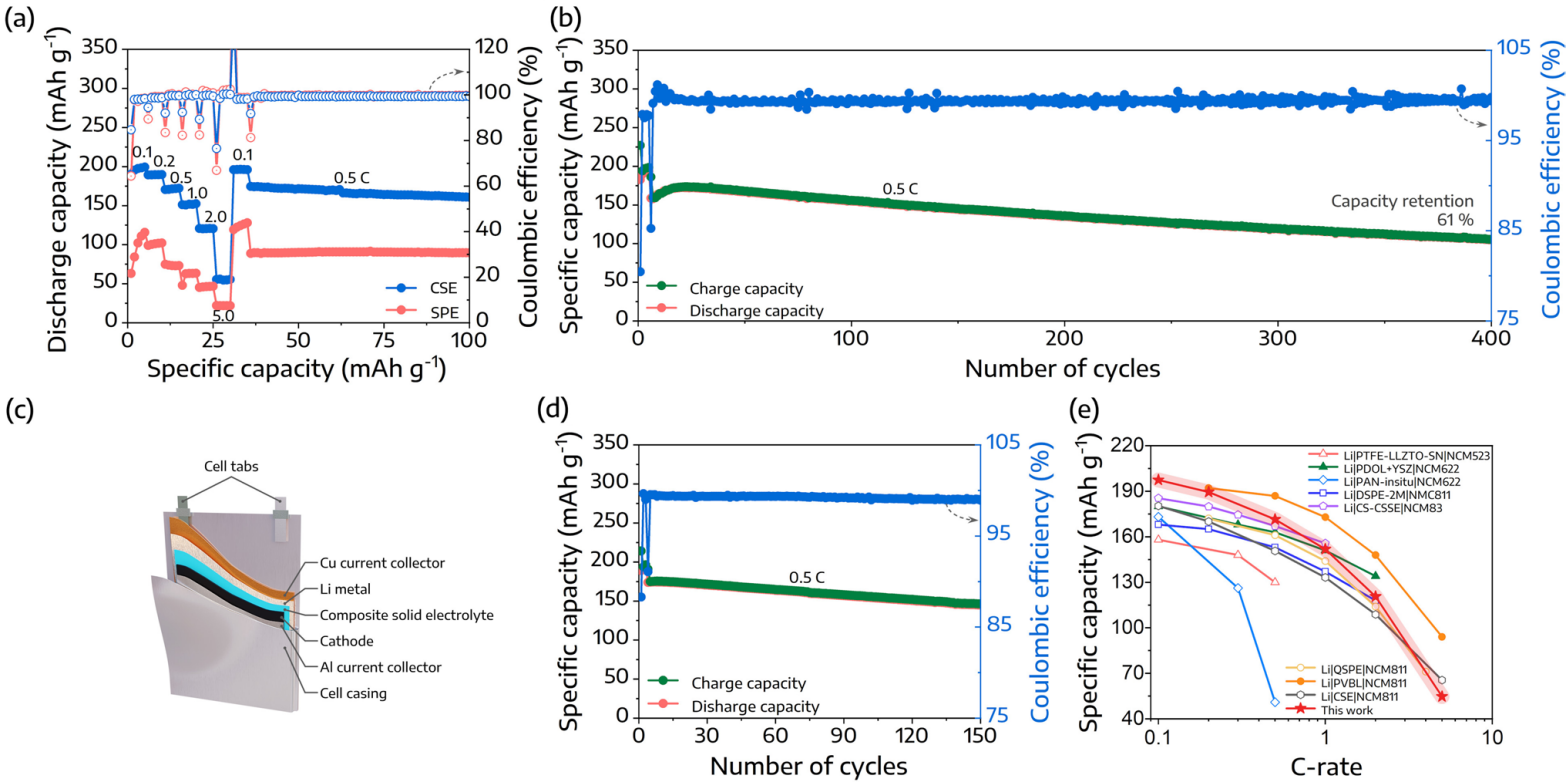
**a** Rate capabilities of the Li|CSE|NCM811 coin cell. **b** Long-term cyclability of the Li|CSE|NCM811 coin cell at 0.5 C and 30 °C. **c** Schematic representation and **d** cyclability of the Li|CSE|NCM811 pouch cell at 0.5 C and 30 °C. **e** Rate capability comparison of recently reported data utilizing an NCM cathode with solid electrolytes at room temperature

The practical applicability of CSEs was further demonstrated by testing full cells composed of a LiNi_0.8_Co_0.1_Mn_0.1_O_2_ (NCM811) cathode and a lithium metal anode at 30 °C. Figures 5a and S8a present the rate capability of the Li|CSE|NCM811 cells, showing discharge capacities of 197.4, 189.4, 171.4, 151.8, 120.6, and 54.7 mAh g^−1^ at rates of 0.1, 0.2, 0.5, 1.0, 2.0, and 5.0 C, respectively. Remarkably, even after experiencing a high rate of 5 C, the capacities recovered to 196.3 and 173.9 mAh g^−1^ upon returning the C-rate to 0.1 C and 0.5 C. On the other hand, the Li|SPE|NCM811 cells displayed significantly lower discharge capacities under equivalent conditions, further reinforcing the superior capacity reversibility and rate capability of CSE.

In addition, the long-term cyclability of Li|CSE|NCM811 is displayed in Fig. 5b. After activation of 5 initial cycles at a low rate of 0.1 C, the cell achieved a discharge-specific capacity of 172.4 mAh g^−1^ at 0.5 C. Furthermore, it maintained a specific reversible capacity of 105.1 mAh g^−1^ after 400 cycles, corresponding to a capacity retention of 61% and an average Coulombic efficiency of 99.4%. Moreover, the Li|CSE|NCM811 cells with the higher mass loadings of NCM811 cathode material, specifically 5.2 and 10.7 mg cm^−2^, were prepared for cyclability testing. As shown in Fig. S8b, following an activation period of 5 cycles at 0.1 C, the Li|CSE|NCM811 cells displayed specific discharge capacities of 163.1 and 107.6 mAh g^−1^ at 0.5 C for mass loadings of 5.2 and 10.7 mg cm^−2^, respectively. After 100 cycles at 0.5 C, these cells still maintained a specific discharge capacity of 116.3 and 65.9 mAh g^−1^, corresponding to capacity retentions of 69.6% and 61.2%, respectively. These observations underscore the impressive long-term cyclability of Li|CSE|NCM811 cells, which is attributed to efficient Li^+^ ion diffusion throughout the cell and uniform lithium deposition on the anode, rendering superior full-cell performance. Furthermore, a single-layer Li|CSE|NCM811 pouch cell was assembled, as shown in Fig. 5c. This cell sustained a discharge capacity of 144.8 mAh g^−1^, with a capacity retention of 83.3% over 150 cycles at 0.5 C (Fig. 5d). These results highlight the promising potential of CSE for scalable applications. Furthermore, the Li|CSE|NCM811 cell displayed a rate capability commensurate with recent data that employed an NCM cathode with solid electrolytes at room temperature, as described in Fig. 5e and summarized in Table S4 [29, 31, 46, 47, 51–54].

**Fig. 6:**
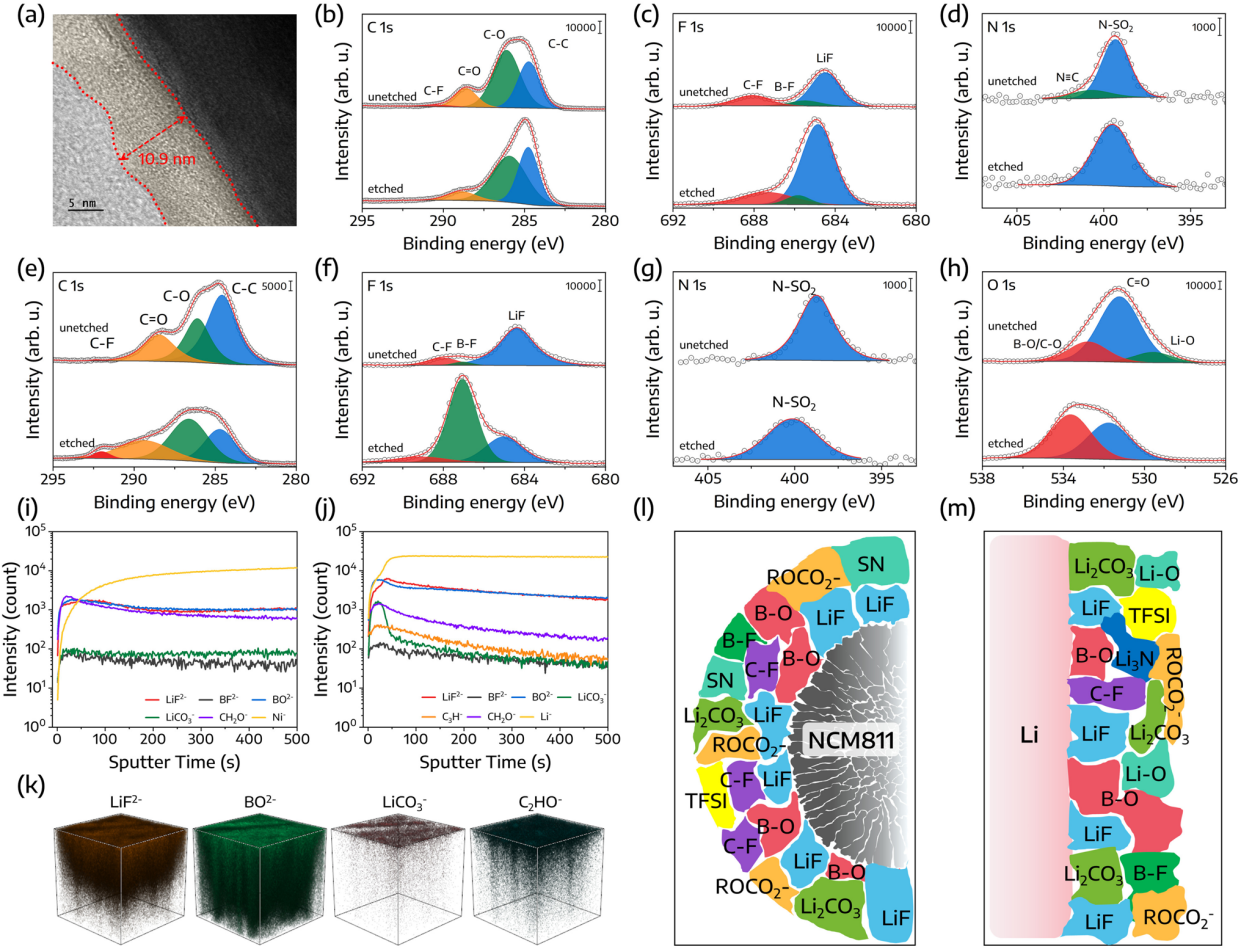
**a** TEM image of the cycled NCM811 electrode. High-resolution XPS spectra of **b** C 1*s*, **c** F 1*s*, and **d** N 1*s* derived from the cycled NCM811. High-resolution XPS spectra of **e** C 1*s*, **f** F 1*s*, **g** N 1*s*, and **h** O 1*s* from the cycled Li anode. ToF–SIMS depth profile of **i** NCM811 cathode and **j** Li anode after cycling and **k** its corresponding 3D spatial distribution. Schematic illustration showing the primary chemical compounds formed on the surface of **l** the cycled NCM811 cathode and **m** the cycled Li anode

The cathode − electrolyte interphase (CEI) formed on cycled NCM811 cathodes was investigated using TEM. As presented in Fig. 6a, the CEI layer exhibited a thickness of approximately 10.9 nm, indicating its capacity to effectively protect the NMC cathode and inhibit further decomposition during cycling. To determine the chemical composition of the CEI, XPS analysis was conducted, as depicted in Figs. 6b–d and S9. The C 1*s* spectrum highlighted a significant enrichment of species such as C–C, C–O, C=O (e.g., ROCO_2_–), and C–F. Concurrently, the F 1*s* spectrum identified the presence of LiF, B–F, and C–F species. The emergence of LiF and B–F suggests that both LiTFSI and LiDFOB undergo decomposition on the NMC surface during cycling. Upon sputtering, the LiF signal was more pronounced, suggesting an enrichment of LiF species within the inner CEI layer. The presence of the C≡N group indicated a thin layer of SN-derived material on the cathode surface, which disappeared post-sputtering [52]. The solid electrolyte interphase (SEI) layer on the cycled Li anode was also scrutinized through XPS analysis, as presented in Figs. 6e–h and S10. The C 1*s* spectrum identified four peaks, which were attributed to C–C, C–O, C=O, and C–F bonds [52, 55]. Following etching, the relative intensity of C–C, C–O, and C=O peaks exhibited a significant reduction, suggesting a polymer-dominated outer SEI layer. Such a malleable outer layer would assist in maintaining intimate contact between the Li metal and solid electrolyte during cycling. In the F 1*s* spectrum, there is a decrease in the intensity of the LiF signal accompanied by an increase in the B–F signal, suggesting that LiF is predominant in the outer SEI layer, while B–F is the main component of the intermediate layer. Moreover, the N–SO_2_ peaks originate from TFSI^–^ in the LiTFSI salt. Additionally, Li–O is detected in the outer layer, while B–O/C–O is dominant in the intermediate layer.

To further decipher the composition and distribution of the CEI and SEI layers, (TOF–SIMS) was executed on the NCM811 and Li metal surfaces after cycling. As demonstrated in Fig. 6i, there is a progressive increase in the intensity of the Ni- signal with increased sputtering time, suggesting the formation of the CEI layer on the cycled NCM811. Furthermore, the outer region manifests an accumulation of signals such as BF^2−^, BO^2−^, LiCO_3_^−^, and CH_2_O^−^, while the inner region exhibits a build-up of LiF^2−^ signals. Similarly, the outer region of the cycled Li anode accumulates signals of BF^2−^, BO^2−^, LiCO_3_^−^, C_3_H^−^, and CH_2_O^−^, while LiF^2−^ signals are concentrated in the inner region (Fig. 6j). The corresponding three-dimensional distribution of LiF^2−^, BO^2−^, LiCO_3_^−^, and CH_2_O^−^ on the Li anode is depicted in Fig. 6k.

Following the XPS and TOF–SIMS analyses, the primary constituents of the CEI and SEI layers are illustrated in Fig. 6l, m, respectively. In summary, it is evident that both sides of the electrode are enriched with LiF- and B-rich species. These constituents serve to prevent direct contact between the electrode surface and the electrolyte, thus reducing interfacial side reactions and enhancing the uniformity and efficiency of Li plating/stripping.

## Conclusion

The CSE was fabricated via the in-situ polymerization of a MEMA-based electrolyte within a self-supported porous LLZO structure. The resulting CSE demonstrated a high ionic conductivity of 1.117 mS cm^−1^ at 30 °C, a substantial lithium transference number of 0.627, and a wide electrochemical window of 5.06 V *vs.* Li/Li^+^. As a result, the Li|CSE|NCM811 cell exhibited remarkable rate capability, facilitating operation at rates of up to 5 C, alongside durable cycling performance, maintaining a discharge-specific capacity of 105.1 mAh g^−1^ following 400 cycles at 0.5 C and 30 °C. This exceptional performance can be ascribed to the synergistic influence of continuous conductive paths facilitated by the self-sustaining porous LLZT backbone, contributing to expedited and efficient Li^+^ diffusion. Additionally, the formation of LiF and B-rich CEI and SEI layers aids in suppressing interfacial side reactions, thereby enhancing the overall performance of SSLMBs. Furthermore, this approach and structure can also be extended to other ceramic, polymer electrolytes, or battery systems. It offers a feasible approach to address the challenges posed by solid electrolytes in the development of safe, high-energy solid-state lithium metal batteries.

## Supplementary Information

Below is the link to the electronic supplementary material.

Supplementary file1 (PDF 880 kb)
