# Supplementary material for: Construction of a High-Performance Composite Solid Electrolyte Through In-Situ Polymerization within a Self-Supported Porous Garnet Framework
Source: Nanomicro Lett. 2024 Jan 12;16:83. doi: 10.1007/s40820-023-01294-0 (PMC10786791; doi:10.1007/s40820-023-01294-0)
Supplement: Supplementary file 1 — Supplementary file1 (PDF 880 kb) [file 40820_2023_1294_MOESM1_ESM.pdf]

Supporting Information for

Construction of a High-Performance Composite Solid Electrolyte through In-Situ Polymerization within a Self-Supported Porous Garnet Framework

An-Giang Nguyen[1], Min-Ho Lee[1], Jaekook Kim[1], and Chan-Jin Park[1,]*

[1]Department of Materials Science and Engineering, Chonnam National University, 77 Yongbong-ro, Buk-gu, Gwangju 61186, South Korea

*Corresponding author. E-mail: parkcj@jnu.ac.kr (Chan-Jin Park)

S1 Supplementary Tables

Table S1 Results of Rietveld refinement analysis for the nominal composition of the self-supported porous $Li_{6.4}La_3Zr_{1.4}Ta_{0.6}O_{12}$ (LLZT)

Structure	Space group	Lattice constant [Å]	Agreement factors [%]			
		$a = b = c$	R_{exp}	R_p	R_{wp}	χ^2
Cubic	$Ia\bar{3}d$	12.9224	8.5338	7.9017	10.7717	1.5932

Atom	Wyckoff site	x	y	z	Occupation
Li(1)	96h	0.062	0.35	0.0.321	0.293
Li(2)	24d	0.375	0	0.25	0.96
La	24c	0.125	0	0.25	1
Zr	16a	0	0	0	0.7
Ta	16a	0	0	0	0.3
O	96h	0.1017	0.1962	0.2816	1

Table S2 Compositions of electrolytes used for ionic conductivity optimization

No	Composition				Ionic conductivity at 30 °C [mS cm^{-1}]
	LiTFSI [mg]	LiDFOB [mg]	SN [mg]	MEMA [mg]	
1	374.9	125.1	250.0	250.0	0.164
2	299.9	100.1	250.0	350.0	0.207
3	187.4	62.6	250.0	500.0	0.450
4	187.4	62.6	350.0	400.0	0.659
5	187.4	62.6	500.0	250.0	0.985

6	262.4	87.6	400.0	250.0	0.699
7	224.9	75.1	250.0	450.0	0.268
8	262.4	87.6	250.0	400.0	0.114
9	337.4	112.6	250.0	300.0	0.163
10	337.4	112.6	300.0	250.0	0.315
11	299.9	100.1	350.0	250.0	0.427
12	224.9	75.1	450.0	250.0	0.840
13	187.4	62.6	450.0	300.0	0.804
14	187.4	62.6	350.0	400.0	0.428
15	187.4	62.6	300.0	450.0	0.428
16	224.9	75.1	400.0	300.0	0.761
17	262.4	87.6	350.0	300.0	0.498
18	224.9	75.1	350.0	350.0	0.378
19	299.9	100.1	300.0	300.0	0.321
20	262.4	87.6	300.0	350.0	0.444
21	224.9	75.1	300.0	400.0	0.442
**22**	**149.9**	**50.1**	**500.0**	**300.0**	**1.117**
23	112.5	37.5	500.0	350.0	0.945
24	75.0	25.0	500.0	400.0	0.493
25	37.5	12.5	500.0	450.0	0.259
26	0.0	0.0	500.0	500.0	0.004
27	149.9	50.1	450.0	350.0	0.849
28	112.5	37.5	450.0	400.0	0.704
29	75.0	25.0	450.0	450.0	0.329
30	37.5	12.5	450.0	500.0	0.128
31	149.9	50.1	400.0	400.0	0.791
32	112.5	37.5	400.0	450.0	0.600
33	75.0	25.0	400.0	500.0	0.292
34	149.9	50.1	350.0	450.0	0.574
35	112.5	37.5	350.0	500.0	0.402
36	75.0	25.0	350.0	550.0	0.205
37	149.9	50.1	300.0	500.0	0.409

38	112.5	37.5	300.0	550.0	0.286
39	75.0	25.0	300.0	600.0	0.246
40	149.9	50.1	250.0	550.0	0.213
41	112.5	37.5	250.0	600.0	0.346
42	75.0	25.0	250.0	650.0	0.143
43	112.5	37.5	550.0	300.0	0.805
45	149.9	50.1	200.0	600.0	0.189
46	224.9	75.1	200.0	500.0	0.200
44	262.4	87.6	500.0	150.0	0.754
47	299.9	100.1	200.0	400.0	0.077
48	112.5	37.5	150.0	700.0	0.102
49	224.9	75.1	150.0	550.0	0.204
50	299.9	100.1	150.0	450.0	0.072
51	337.4	112.6	150.0	400.0	0.044
52	187.4	62.6	100.0	650.0	0.104
53	299.9	100.1	100.0	500.0	0.043
54	262.4	87.6	50.0	600.0	0.052
55	187.4	62.6	0.0	750.0	0.039
56	262.4	87.6	0.0	650.0	0.026
57	374.9	125.1	0.0	500.0	0.005
58	187.4	62.6	400.0	350.0	0.738
59	299.9	100.1	400.0	200.0	0.656
60	374.9	125.1	200.0	300.0	0.111

Table S3 Quantification of Li^+ local environment in CSE before and after testing

**Sample**		**Li^+ local environment**		
		LLZT	Polymer	Interface
Before	Area	8378.20	1663.38	869.68
	Percent [%]	76.79	15.24	7.97
After	Area	57594.09	16703.65	27115.25
	Percent [%]	56.79	16.47	26.74
Increase factor		6.87	10.04	31.18

Table S4 Comparison of the electrochemical performances of solid electrolytes when using NCM cathode at room temperature

**Config.**	**C-rate and corresponding specific capacity** [mAh g^{-1}]								
	0.1C	0.2C	0.3C	0.5C	1C	2C	3C	4C	5C
Li|PTFE-LLZTO-SN|NCM523 [S1]	158	–	148	130	–	–	–	–	–
Li|PAN-insitu|NCM622 [S2]	173.1	–	126.2	51	–	–	–	–	–
Li|QSPE|NMC811 [S3]	–	172	–	161	144	114	–	71	–
Li|DSPE-2M|NMC811 [S4]	168	165	–	153	137	118	–	–	–
Li|CSE|NCM811 [S5]	180.3	170	–	150.7	133.2	108.8	–	–	65.5
Li|PVBL|NCM811 [S6]	–	192.1	–	186.9	172.9	147.9	–	–	94
Li|PDOL+YSZ|NCM622 [S7]	180	–	168	163	151	134	–	–	–
Li|CS-CSSE| NCM83 [S8]	185.4	179.9	174.5	167	155.7		–	–	–
This work	197.4	189.4	–	171.4	151.8	120.6	–	–	54.7

S2 Supplementary Figures

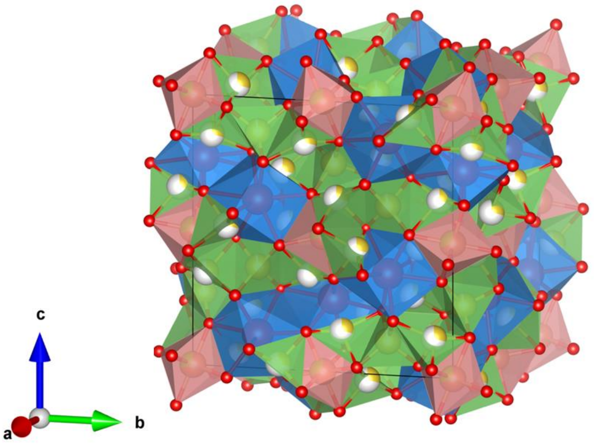

Fig. S1 Illustration of the crystal structure of self-supported porous LLZT

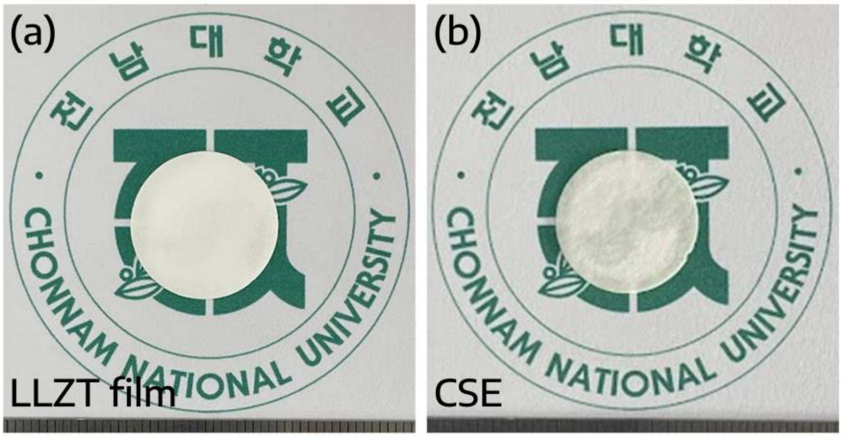

Fig. S2 Optical photographs of the self-supported porous LLZT and CSE films

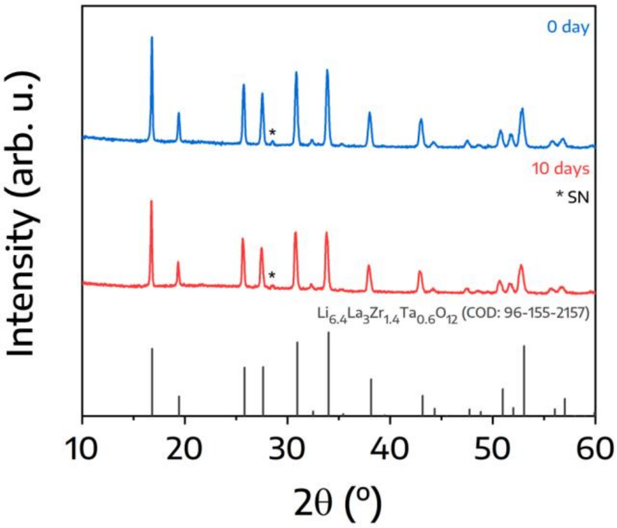

Fig. S3 XRD patterns of CSE before and after 10 days of exposure to the ambient environment

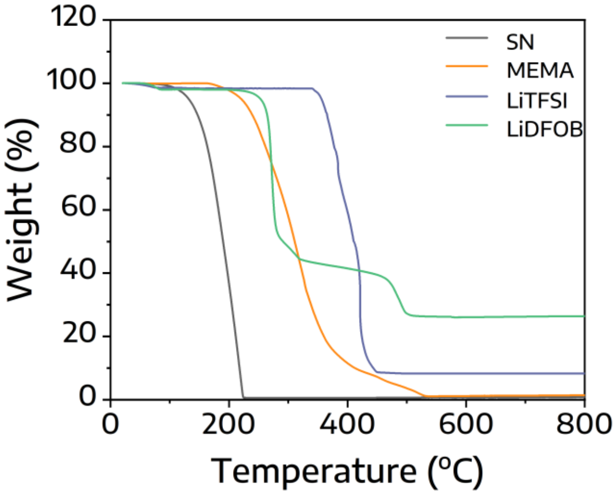

Fig. S4 TGA curves of SN, MEMA, LiTFSI, and LiDFOB in an air atmosphere

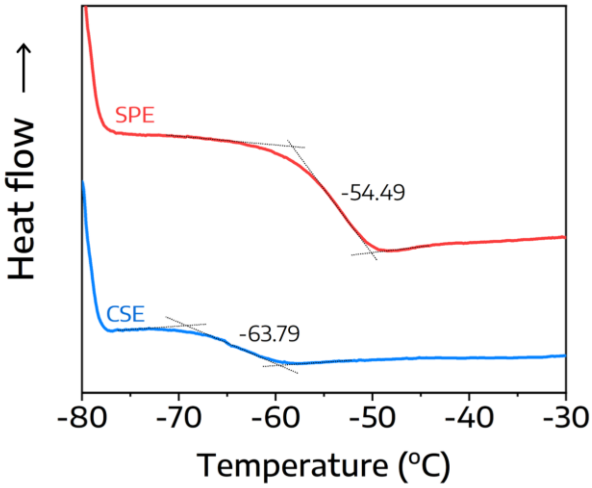

Fig. S5 DSC profiles of SPE and CSE

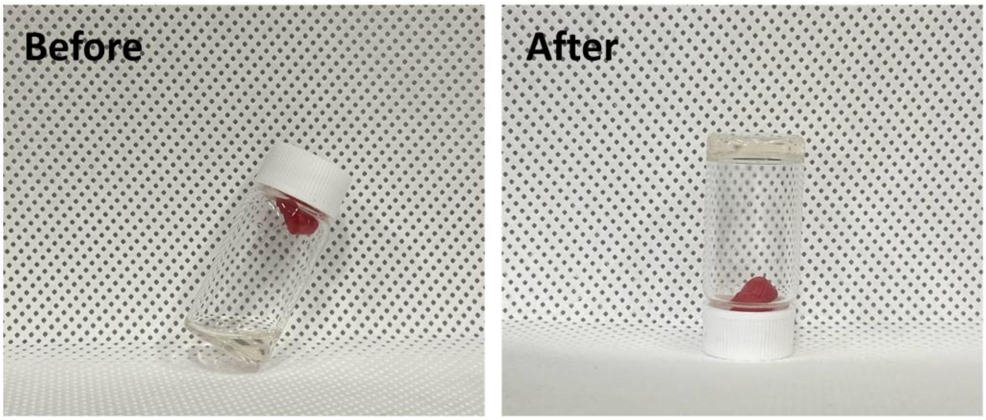

Fig. S6 Photographs of the monomer solution before and after polymerization

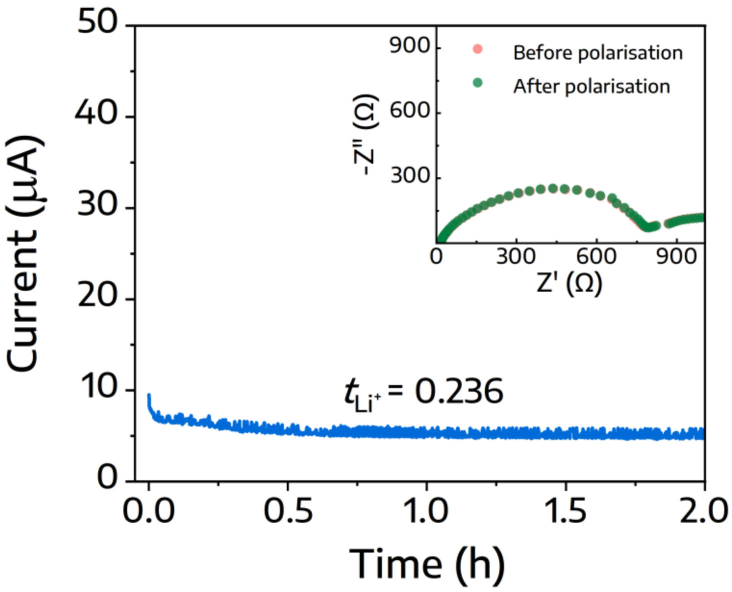

Fig. S7 Current transient profile and the corresponding EIS plots of Li|SPE|Li symmetric cell before and after polarization

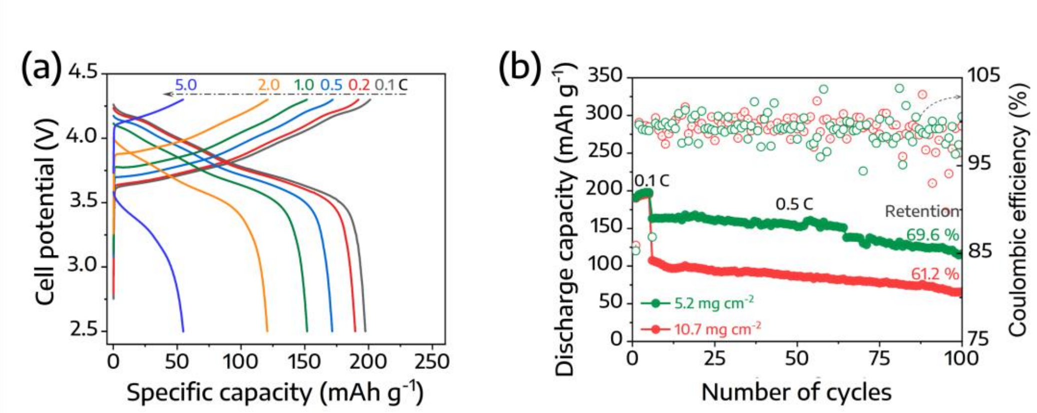

Fig. S8 (**a**) Galvanostatic charge-discharge profiles at various C-rates of the Li|CSE|NCM811 coin cell. (**b**) Cyclability of Li|CSE|NCM811 coin cells using NCM811 with high mass loadings of 5.2 and 10.7 mg cm^{-2}

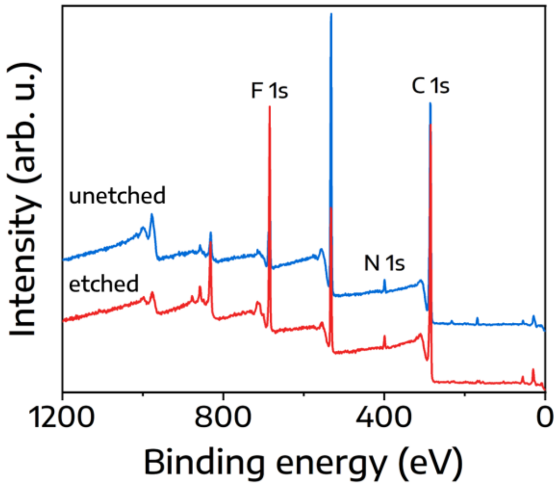

Fig. S9 Wide-range XPS spectrum of cycled NCM811 cathode

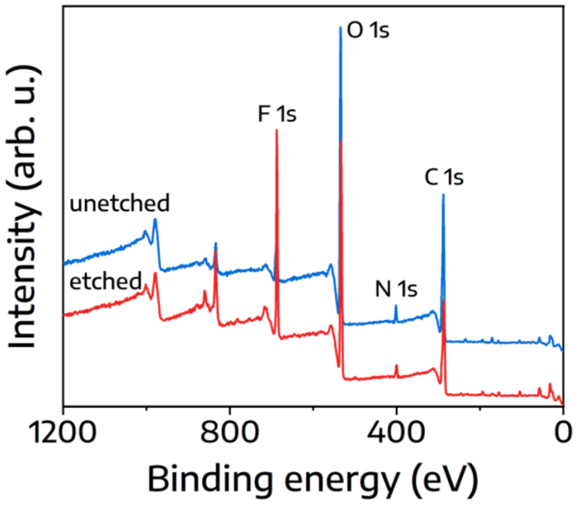

Fig. S10 Wide-range XPS spectrum of cycled Li anode

Supplementary References

[S1] T.L. Jiang, P.G. He, G.X. Wang, Y. Shen, C.W. Nan et al., Solvent-free synthesis of thin, flexible, nonflammable garnet-based composite solid electrolyte for all-solid-state lithium batteries. Adv. Energy Mater. **10**(12), 1903376 (2020). https://doi.org/10.1002/aenm.201903376

[S2] M. Yao, Q.Q. Ruan, T.H. Yu, H.T. Zhang, S.J. Zhang, Solid polymer electrolyte with in-situ generated fast Li+ conducting network enable high voltage and dendrite-free lithium metal battery. Energy Storage Mater. **44**, 93-103 (2022). https://doi.org/10.1016/j.ensm.2021.10.009

[S3] J. Yu, X.D. Lin, J.P. Liu, J.T.T. Yu, M.J. Robson et al., In situ fabricated quasi-solid polymer electrolyte for high-energy-density lithium metal battery capable of subzero operation. Adv. Energy Mater. **12**(2), 2102932 (2022). https://doi.org/10.1002/aenm.202102932

[S4] X.D. Lin, J. Yu, M.B. Effat, G.D. Zhou, M.J. Robson et al., Ultrathin and non-flammable dual-salt polymer electrolyte for high-energy-density lithium-metal battery. Adv. Funct. Mater. **31**(17), 2010261 (2021). https://doi.org/10.1002/adfm.202010261

[S5] A.G. Nguyen, R. Verma, G.C. Song, J. Kim, C.J. Park, In situ polymerization on a 3D ceramic framework of composite solid electrolytes for room-temperature solid-state batteries. Adv. Sci., 2207744 (2023). https://doi.org/10.1002/advs.202207744

[S6] P.R. Shi, J.B. Ma, M. Liu, S.K. Guo, Y.F. Huang et al., A dielectric electrolyte composite with high lithium-ion conductivity for high-voltage solid-state lithium metal batteries. Nat. Nanotechnol. **18**(6), 602 (2023). https://doi.org/10.1038/s41565-023-01341-2

[S7] H. Yang, B. Zhang, M.X. Jing, X.Q. Shen, L. Wang et al., In situ catalytic polymerization of a highly homogeneous PDOL composite electrolyte for long-cycle high-voltage solid-state lithium batteries. Adv. Energy Mater. **12**(39), 2201762 (2022). https://doi.org/10.1002/aenm.202201762

[S8] C.S. Bao, C.J. Zheng, M.F. Wu, Y. Zhang, J. Jin et al., 12 mu m-thick sintered garnet ceramic skeleton enabling high-energy-density solid-state lithium metal batteries. Adv. Energy Mater. **13**(13), 2204028 (2023). https://doi.org/10.1002/aenm.202204028
